# Predicting academic career outcomes by predoctoral publication record

**DOI:** 10.7717/peerj.5707

**Published:** 2018-10-04

**Authors:** Jason R. Tregellas, Jason Smucny, Donald C. Rojas, Kristina T. Legget

**Affiliations:** 1 Department of Psychiatry, University of Colorado School of Medicine, Aurora, CO, USA; 2 Research Service, VA Medical Center, Denver, CO, USA; 3 Department of Psychology, Colorado State University, Fort Collins, CO, USA

**Keywords:** Academic career outcomes, Faculty positions, Predoctoral publication record, Biomedical PhD programs, Education, Graduate education

## Abstract

**Background:**

For students entering a science PhD program, a tenure-track faculty research position is often perceived as the ideal long-term goal. A relatively small percentage of individuals ultimately achieve this goal, however, with the vast majority of PhD recipients ultimately finding employment in industry or government positions. Given the disparity between academic career ambitions and outcomes, it is useful to understand factors that may predict those outcomes. Toward this goal, the current study examined employment status of PhD graduates from biomedical sciences programs at the University of Colorado Anschutz Medical Campus (CU AMC) and related this to metrics of predoctoral publication records, as well as to other potentially important factors, such as sex and time-since-degree, to determine if these measures could predict career outcomes.

**Methods:**

Demographic information (name, PhD program, graduation date, sex) of CU AMC biomedical sciences PhD graduates between 2000 and 2015 was obtained from University records. Career outcomes (academic faculty vs. non-faculty) and predoctoral publication records (number and impact factors of first-author and non-first-author publications) were obtained via publicly available information. Relationships between predoctoral publication record and career outcomes were investigated by (a) comparing faculty vs. non-faculty publication metrics, using *t*-tests, and (b) investigating the ability of predoctoral publication record, sex, and time-since-degree to predict career outcomes, using logistic regression.

**Results:**

Significant faculty vs. non-faculty differences were observed in months since graduation (*p* < 0.001), first-author publication number (*p* = 0.001), average first-author impact factor (*p* = 0.006), and highest first-author impact factor (*p* = 0.004). With sex and months since graduation as predictors of career outcome, the logistic regression model was significant (*p* < 0.001), with both being male and having more months since graduation predicting career status. First-author related publication metrics (number of publications, average impact factor, highest impact factor) all significantly improved model fit (χ^2^ < 0.05 for all) and were all significant predictors of faculty status (*p* < 0.05 for all). Non-first-author publication metrics did not significantly improve model fit or predict faculty status.

**Discussion:**

Results suggest that while sex and months since graduation also predict career outcomes, a strong predoctoral first-author publication record may increase likelihood of obtaining an academic faculty research position. Compared to non-faculty, individuals employed in faculty positions produced more predoctoral first-author publications, with these being in journals with higher impact factors. Furthermore, first-author publication record, sex, and months since graduation were significant predictors of faculty status.

## Introduction

The journey to the successful completion of a PhD is a long and arduous one, with the median time to complete a science or engineering doctorate from U.S. Universities in 2016 being 6.6 years (National Science Foundation & National Center for Science and Engineering Statistics, 2018a). As such, understanding the array of post-PhD likely career outcomes and myriad factors that may contribute to these different paths is critical, not only for individuals considering or already engaged in a graduate program, but also for the educators and faculty mentors guiding new generations of scientists. Obtaining this information is often difficult, however, as few PhD-granting institutions track their graduates’ employment outcomes. While efforts are currently underway to increase reporting of this information, such as the Coalition for Next Generation Life Science (Blank et al., 2017), transparency regarding student outcomes is lacking for most PhD programs at present.

For individuals entering into a science PhD program, a career in academic research is often perceived as the ideal long-term goal. In this context, a tenured or tenure-track faculty research position is often considered the gold standard. Despite this, a relatively small percentage of individuals successfully completing a PhD ultimately achieve this goal, with only an estimated 14% of biological sciences PhD recipients having a tenure-track faculty position 5–6 years post graduation (Stephan, 2015). This rate is somewhat higher for earners of chemistry (23%) or physics (21%) PhDs.

The disparity between ambitions of a faculty research position and actual outcomes also was illustrated by a 2015 Nature global survey of 3,451 science graduate students, investigating student perceptions about their graduate school experiences and future career opportunities (Woolston, 2015). The survey found that 78% of students reported they were “likely” or “very likely” to pursue a research career in academia. This figure likely reflects the opinion, as gathered from a 2010 survey of 4,109 PhD students, that a faculty research career is the most attractive career path, compared to careers in teaching, government, established or startup firms, or other settings (Sauermann & Roach, 2012). While some studies have suggested that the appeal of an academic research career may decrease somewhat as students progress through graduate school, this path remains the most favored career goal (Sauermann & Roach, 2012; Roach & Sauermann, 2017). Despite academia’s apparent desirability, however, the vast majority of PhD recipients ultimately find employment in industry, government, or other science or non-science-focused teaching, writing, or business settings.

With such an imbalance between PhD students desiring academic research positions and the availability of these positions, it is useful to examine various factors that may serve as predictors of the likelihood of students achieving these goals. As such, this study examined the employment status of PhD graduates from biomedical sciences programs at the University of Colorado Anschutz Medical Campus (CU AMC) from 2000 to 2015, and related these outcomes to publicly available metrics of the students’ graduate school activities, namely factors related to the publication of peer-reviewed manuscripts, which are often weighted heavily as indicators of academic research success. Our primary hypothesis was that a higher number of first-author publications and higher publication journal impact factors would predict students’ later ability to secure faculty academic research positions. The impact of time-since-degree on career outcomes was also examined, as longer postdoctoral training periods may be associated with eventual tenure-track academic research positions, with shorter training periods associated with non-academic careers (National Institutes of Health, 2012). Correspondingly, the average age at which postdoctoral fellows are hired as faculty is greater than the age at which they are hired as non-faculty (National Institutes of Health, 2012). Additionally, the influence of sex on career outcomes was assessed, due to previous observations of differences in the distribution of males and females in academic faculty positions in the biomedical sciences. That is, males are over-represented in academic faculty positions (Sheltzer & Smith, 2014; Jena et al., 2015; McDermott et al., 2018).

## Materials and Methods

### Data collection

Demographic information (name, PhD program, date of graduation, and sex) of PhD graduates from biomedical sciences programs at CU AMC from the years 2000–2015 was obtained from records provided by the University. Graduates of the following current and former programs were included: Biochemistry and Molecular Genetics; Biophysics and Genetics; Cell and Developmental Biology; Cell Biology, Stem Cells, and Development; Human Medical Genetics; Immunology; Microbiology; Microbiology and Immunology; Molecular Biology; Neuroscience; Pathology; Pharmacology; Physiology. MD-PhD students were not included. Individuals who graduated fewer than 24 months prior to data collection were excluded (as data were collected in May 2015, students graduating May 2013 or later were not included). None of the individuals graduating May 2013 or later had achieved our definition of tenure-track or equivalent faculty status (see below) as of the time of data collection. In total, 363 individuals were included in the analysis.

Using this information, occupational history, predoctoral publication history, and predoctoral publication impact factors were obtained for each individual using publicly available databases and search engines (Google Search, Google Scholar, LinkedIn, ResearchGate, PubMed; 2-year journal impact factors obtained from the 2014 *Journal Citation Reports* database (Clarivate Analytics, 2015)). “Predoctoral” publications were defined by the following criteria: (1) publication date no greater than 1 year after graduation date, to preclude postdoctoral publications, (2) publication date after PhD program entry date, and (3) at least one author in addition to the student being affiliated with CU AMC (formerly University of Colorado Denver).

Occupational history was classified using a binary criterion, in which individuals who are current or former faculty members at PhD-granting institutions were classified as having obtained academic tenure-track or equivalent faculty positions, that is, Assistant, Associate or Full Professor (henceforth abbreviated as “faculty members”), and all other individuals classified otherwise (“non-faculty members”). Research Assistant Professors were not considered “faculty members,” nor were Instructors, Adjunct Professors, Research Associates, Research Technicians, Postdoctoral Fellows, faculty in liberal arts colleges or non-PhD-granting institutions, scientists in private pharmaceutical or government institutions, or individuals who pursued alternative careers. Individuals who could not be located using standard search engines were also not considered tenure-track or equivalent faculty, as we deemed it unlikely that such individuals held these positions.

All methods were approved by the Colorado Multiple Institutional Review Board (COMIRB Protocol 14-2024). Informed consent was not required, as the study utilized existing data and was approved by COMIRB as exempt. Data are from the University of Colorado Denver, Office of the Registrar. The University of Colorado Denver, Office of the Registrar has approved this publication.

### Data analysis

We analyzed the relationship between predoctoral publication record and future faculty status using two methods, in SPSS v.23 (IBM Corp., Armonk, NY, USA). First, we simply compared publication metrics between faculty members and non-faculty members (total first-author publications, total non-first-author publications, average impact factor of first-author publications, average impact factor of non-first-author publications, highest impact factor of first-author publications, and highest impact factor of non-first-author publications) using two-tailed *t*-tests, with a Bonferroni-corrected alpha level of 0.008 (i.e., 0.05/6).

Our primary analysis examined the ability of potentially important demographic information (sex; time since degree) and predoctoral publication record to predict career outcomes using logistic regression. A baseline model was first constructed in which sex and months since graduation were entered as predictors. This was motivated by previous findings showing (1) a significant difference in the distribution of males and females in academic faculty positions in the biomedical sciences (males > females) (Sheltzer & Smith, 2014; Jena et al., 2015; McDermott et al., 2018), and (2) the average training period length for postdoctoral fellows eventually hired as faculty is greater than the training period for those hired as non-faculty (National Institutes of Health, 2012). The ability of various publication-related metrics (number of first-author publications, number of non-first-author publications, average impact factor of first-author publications, average impact factor of non-first-author publications, highest impact factor of first-author publications, highest impact factor of non-first-author publications) to significantly improve model fit above the baseline model was then evaluated by Chi-squared tests. Number and impact factor of publications were also combined into single composite measures (log [sum impact factor of publications +1]), separately for first- and non-first-author publications, which were also evaluated for ability to predict faculty status. The goal of these composite measures was to provide a weighted count of publications (weighted by impact factor) that may better predict outcomes and would negate potential issues related to the collinearity of the other measures. The term was increased by a value of one to avoid omitting individuals with zero publications. Accuracy estimates were weighted by the ratio of faculty/non-faculty members (12%). This adjustment was made to avoid inflating specificity at the expense of sensitivity. As 88% of the individuals in the sample were non-faculty, without weighting by faculty/non-faculty ratio, the logistic regression model would always predict an individual to be non-faculty (even in the absence of any predictors) and consequently achieve 88% accuracy. With weighting, the “empty” model is “normalized” to 50% accuracy.

To further explore our findings, we tested the ability of support vector machine (SVM; Matlab 2014b Statistics and Machine Learning Toolbox) classifiers to predict faculty status using the sets of predictors examined by logistic regression. Classifiers were trained in a *k*-fold (*k* = 10) cross-validation based framework, in which nine of the 10 folds were used for training and the remaining fold for testing. Because the folds were allocated at random, the process was repeated 100 times and the performance metrics of interest averaged for all repetitions. Folds were weighted according to faculty/non-faculty ratio to ensure that all profiles were sufficiently represented in each fold during training.

## Results

Predoctoral publication data for biomedical science PhD graduates who had obtained tenure-track or equivalent academic faculty positions at PhD-granting institutions at the time of study (“faculty members”; *n* = 40) and those with other career outcomes (“non-faculty members”; *n* = 323) are presented in Table 1. Significant differences were observed between faculty and non-faculty for sex (M/F ratio = 24/16 for faculty, 137/186 for non-faculty, χ^2^ = 4.46, *p* = 0.035, odds ratio = 2.04), months since graduation (*t* (361) = 5.42, *p* < 0.001, *d* = 0.97) total number of first-author publications (*t* (361) = 3.25, *p* = 0.001, *d* = 0.55), average impact factor of first-author publications (*t* (361) = 2.77, *p* = 0.006, *d* = 0.46), and highest impact factor of first-author publications (*t* (361) = 2.87, *p* = 0.004, *d* = 0.42). No significant group differences were observed for non-first-author publication-related metrics.

**Table 1:** Participant publication information.

Measure	Group	Mean	SE	Effect size (*d*)
***Months since graduation***	Faculty	125.55	5.71	0.97
	Non-faculty	87.33	2.38	
***First author publications***	Faculty	2.25	0.18	0.55
	Non-faculty	1.63	0.064	
***Non-first-author publications***	Faculty	1.63	0.24	0.06
	Non-faculty	1.72	0.097	
***Average impact factor (first-author publications)***	Faculty	7.15	0.70	0.46
	Non-faculty	5.10	0.25	
***Average impact factor (non-first-author publications)***	Faculty	4.87	0.63	0.14
	Non-faculty	5.60	0.36	
***Highest impact factor (first-author publications)***	Faculty	9.52	1.39	0.42
	Non-faculty	6.34	0.35	
***Highest impact factor (non-first-author publications)***	Faculty	7.03	1.45	0.04
	Non-faculty	7.40	0.51	

**Figure 1:**
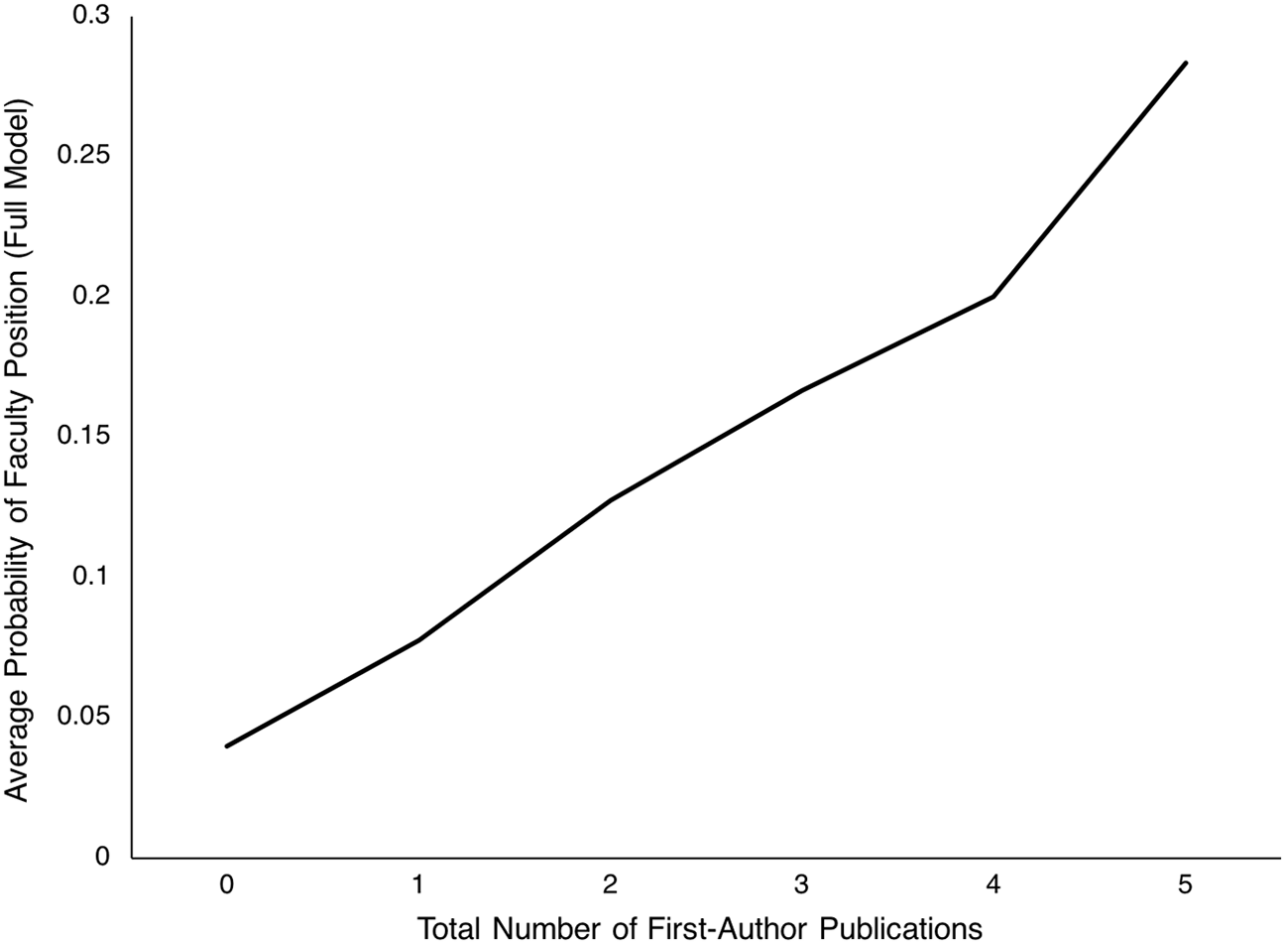
Probability of obtaining a faculty position as a function of total number of first-author publications.

Demographic and publication metrics were subsequently used in logistic regression analyses to derive predictive models of faculty status. First, sex and months since graduation were entered as predictors in a baseline model. Using these predictors, the model was significant, as both being male and having more months since graduation predicted faculty status (Table 2). The logistic regression baseline model showed 69% accuracy, driven by high negative predictive values, but low positive predictive values (Table 3). In other words, high negative and low positive predictive values indicate that sex and months since graduation can predict with high accuracy those who *will not* obtain faculty positions, but with low accuracy who *will* obtain a faculty position. Predoctoral publication metrics were then individually added to the baseline model and evaluated (see Materials and Methods). First-author-related publication metrics (number of first-author publications, average impact factor of first-author publications, highest impact factor of first-author publications) all significantly improved model fit (step [vs. baseline] χ^2^ < 0.05 for all) and were all significant predictors of faculty status (Table 2). An illustration of the most predictive of these factors, number of first-author publications, is shown in Fig. 1. The plot demonstrates a fairly linear relationship between number of publications and probability of obtaining a faculty position. Non-first-author-related metrics, however, did not significantly improve model fit or predict faculty status (Table 2). Log-likelihood and fit characteristics (Akaike and Bayesion information criteria) further suggested the best-fitting models included first-author-related metrics (Table 2). A measure that combined number and impact factor of first-author publications (weighted first-author publication count) also significantly predicted faculty status (Table 2). A plot of the relationship between this metric and probability of obtaining a faculty position can be found in Fig. S1. Notably, including first-author related measures (particularly those relating to impact factors) decreased the impact of sex on predicting faculty status (e.g., smaller beta weight and *p*-value was observed for the sex predictor in the baseline model 1 vs. model 1 g in Table 2). Model accuracies ranged from 69% to 73%, depending on the model (Table 3). These results were primarily driven by high negative predictive values 95–96%, but low positive predictive values (23–26%). Similar accuracies were observed using SVM classifiers (Table 4).

**Table 2:** Logistic regression model comparison.

Model	Predictors	Model χ^2^ (*p)*	Step** χ^2^ (*p*)	−2 LL	AIC	BIC	C–S *R*^2^	Nagel *R*^2^	Predictor			Predictor OR {95% CI}
									B	SE	*p*	
**1 (Baseline)**	• *Constant*	34.23 (<0.001)	–	217.64	223.64	225.32	0.09	0.18	−4.08	0.57	<0.001	0.02 {–}
	• Sex*								−0.93	0.37	0.01	0.40 {0.19–0.81}
	• *Months since graduation*								0.02	0.01	<0.001	1.02 {1.01–1.03}
**1a**	• *Constant*	45.16 (<0.001)	***10.94 (0.001)***	206.70	214.70	216.94	0.12	0.23	−5.14	0.69	<0.001	0.01 {–}
	• Sex*								−0.98	0.38	0.01	0.37 {0.18–0.79}
	• *Months since graduation*								0.02	0.01	<0.001	1.02 {1.02–1.03}
	• ***Total first-author publications***								***0.49***	***0.15***	***0.001***	***1.64 {1.22–2.20}***
**1b**	• *Constant*	34.64 (<0.001)	***0.41 (0.52)***	217.22	225.22	227.46	0.09	0.18	−3.97	0.59	<0.001	0.02
	• Sex*								−0.94	0.37	0.01	0.39 {0.19–0.80}
	• *Months since graduation*								0.02	0.01	<0.001	1.02 {1.01–1.03}
	• ***Total non-first-author publications***								−***0.07***	***0.11***	***0.53***	***0.93 {0.75–1.16}***
**1c**	• *Constant*	34.23 (<0.001)	***4.73 (0.03)***	212.91	220.91	223.15	0.10	0.20	−4.66	0.67	<0.001	0.01 {−}
	• Sex*								−0.79	0.37	0.03	0.45 {0.22–0.94}
	• *Months since graduation*								0.02	0.01	<0.001	1.02 {1.01–1.03}
	• ***Average impact factor first-author publications***								***0.08***	***0.03***	***0.02***	***1.08 {1.01–1.15}***
**1d**	• *Constant*	35.97 (<0.001)	***1.75 (0.19)***	215.89	223.89	226.13	0.09	0.19	−3.90	0.59	<0.001	0.02 {−}
	• Sex*								−0.97	0.37	0.01	0.38 {0.19–0.78}
	• *Months since graduation*								0.02	0.01	<0.001	1.02 {1.01–1.03}
	• ***Average impact factor non-first-author publications***								−***0.04***	***0.03***	***0.23***	***0.96 {0.90–1.03}***
**1e**	• *Constant*	39.09 (<0.001)	***4.87 (0.027)***	212.77	220.77	223.01	0.10	0.20	−4.56	0.64	<0.001	0.01 {−}
	• Sex*								−0.81	0.37	0.03	0.45 {0.22–0.92}
	• *Months since graduation*								0.02	0.01	<0.001	1.02 {1.01–1.03}
	• ***Highest impact factor first-author publications***								***0.05***	***0.02***	***0.02***	***1.05 {1.01–1.09}***
**1f**	• *Constant*	35.00 (<0.001)	***0.77 (0.38)***	216.87	224.87	227.11	0.09	0.18	−3.98	0.58	<0.001	0.02 {−}
	• Sex*								−0.96	0.37	0.01	0.38 {0.19–0.79}
	• *Months since graduation*								0.02	0.01	<0.001	1.02 {1.01–1.03}
	• ***Highest impact factor non-first-author publications***								−***0.02***	***0.02***	***0.40***	***0.98 {0.95–1.02}***
**1g**	• *Constant*	48.92 (<0.001)	***14.70 (<0.001)***	202.94	210.94	213.18	0.13	0.25	−6.03	0.87	<0.001	0.00 {−}
	• Sex*								−0.83	0.38	0.03	0.43 {0.21–0.91}
	• *Months since graduation*								0.02	0.01	<0.001	1.02 {1.01–1.03}
	• ***Weighted first-author publication count***								***0.79***	***0.23***	***0.001***	***2.21 {1.41–3.46}***
**1h**	• *Constant*	34.69 (<0.001)	***0.47 (0.49)***	217.79	225.79	228.03	0.09	0.18	−3.91	0.62	<0.001	0.02 {−}
	• Sex*								−0.94	0.37	0.01	0.39 {0.19–0.80}
	• *Months since graduation*								0.02	0.01	<0.001	1.02 {1.01–1.03}
	• ***Weighted non-first-author publication count***								−***0.09***	***0.14***	***0.49***	***0.91 {0.70–1.19}***

**Notes:**

AIC, Akaike Information Criterion; BIC, Bayesian Information Criterion; B, Beta; C–S, Cox and Snell; LL, log likelihood; Nagel, Nagelkerke; OR, odds Ratio; SE, standard error.

* Negative B values for the sex predictor imply greater odds of being a faculty member in men vs. women.

** Step values are for baseline model vs. an additional predictor (written in bold italic), for example, Total first-author publications in Model 1a.

**Table 3:** Predictive metrics (%) for each model calculated by logistic regression.

Model	Predictors	Specificity	Sensitivity	PPV	NPV	Accuracy
**1 (Baseline)**	Constant Sex Months since graduation	69	75	23	96	69
**1a**	Constant Sex Months since graduation ***Total first-author publications***	73	75	26	96	73
**1b**	Constant Sex Months since graduation ***Total non-first-author publications***	71	73	24	95	71
**1c**	Constant Sex Months since graduation ***Average impact factor first-author publications***	71	75	24	96	71
**1d**	Constant Sex Months since graduation ***Average impact factor non-first-author publications***	71	75	24	96	72
**1e**	Constant Sex Months since graduation ***Highest impact factor first-author publications***	71	78	25	96	71
**1f**	Constant Sex Months since graduation ***Highest impact factor non-first-author publications***	72	73	24	95	72
**1g**	Constant Sex Months since graduation ***Weighted first-author publication count***	73	73	25	96	73
**1h**	Constant Sex Months since graduation ***Weighted non-first-author publication count***	70	75	24	96	71

**Notes:**

PPV, positive predictive value; NPV, negative predictive value. “Positive” status for PPV was being a tenure-track faculty member.

**Table 4:** Predictive metrics (%) for each model calculated using support vector machines.

Model	Predictors	Specificity	Sensitivity	PPV	NPV	Accuracy
**1 (Baseline)**	Sex Months since graduation	65	78	21	96	66
**1a**	Sex Months since graduation ***Total first-author publications***	69	78	24	96	70
**1b**	Sex Months since graduation ***Total non-first-author publications***	65	79	22	96	66
**1c**	Sex Months since graduation ***Average impact factor first-author publications***	65	77	22	96	66
**1d**	Sex Months since graduation ***Average impact factor non-first-author publications***	66	80	23	96	68
**1e**	Sex Months since graduation ***Highest impact factor first-author publications***	64	77	21	96	66
**1f**	Sex Months since graduation ***Highest impact factor non-first-author publications***	65	75	21	96	66
**1g**	Sex Months since graduation ***Weighted first-author publication count***	71	73	24	96	71
**1h**	Sex Months since graduation ***Weighted non-first-author publication count***	65	79	22	96	66

**Notes:**

PPV, positive predictive value; NPV, negative predictive value. “Positive” status for PPV was being a tenure-track faculty member.

## Discussion

As hypothesized, significant differences were observed between predoctoral first-author publication records of academic faculty vs. non-faculty. Specifically, compared to non-faculty, faculty produced more predoctoral first-author publications. Additionally, faculty predoctoral first-author publications were in journals with higher impact factors. Significant predictors of faculty vs. non-faculty status included sex, months since graduation, and first-author publication record. As a caveat, however, the best models were all less than 75% accurate, suggesting that variables other than those considered here are also likely to be important factors in predicting future faculty status. Importantly, negative predictive value was high, while positive predictive value was low, suggesting high accuracy in predicting those that will *not* obtain a faculty position, but poor accuracy in predicting those who *will* obtain a faculty position. This pattern may suggest that while first-author publications are necessary for obtaining a faculty position, they are not sufficient. Plotting the probability of obtaining a faculty position as a function of total number of first-author publications also revealed a strikingly linear relationship between the two metrics. This curve did not plateau, suggesting that stronger first-author publication records than examined in this study may continue to improve the probability of future faculty employment.

The present results are consistent with a previous finding demonstrating a relationship between first-author publication record during the first 8 years after the first publication and eventual principal investigator status (Van Dijk, Manor & Carey, 2014), which would largely align with our definition of faculty status. Because the previous study focused on all publications prior to becoming a principal investigator, including both predoctoral and postdoctoral work, the current findings add that predoctoral publication record in itself may be a useful predictor, as we attempted to exclude postdoctoral publications. Interestingly, however, a model that only included sex and months since graduation was also a fair predictor of future faculty status. As men are over-represented in faculty positions (Sheltzer & Smith, 2014; Jena et al., 2015; McDermott et al., 2018) and the average biomedical science postdoctoral fellowship has increased in length from 1–2 to 4 or more years (National Institutes of Health, 2012), this result is not entirely surprising.

Several factors may account for limitations of the full final model in predicting faculty status, including (1) choosing alternative pathways to academia regardless of publication record, (2) variable academic job markets, and (3) extraneous circumstances, for example, personal factors, such as poor health or geographic restrictions. It is also possible that the competitiveness of the academic job market, in which hundreds of applicants frequently apply for each faculty position, makes strong publication records necessary but not sufficient for hiring. Nonetheless, our results do suggest that a strong predoctoral first-author publication record may increase the likelihood of obtaining an academic faculty research position. A potential limitation of the current investigation is that the relationship between predoctoral publication record and subsequent postdoctoral appointment was not assessed. This is likely another key factor in a later transition to a faculty position. Furthermore, the scope of the current investigation did not allow determination of the influence of postdoctoral publication record itself on subsequent faculty status. As such, future studies investigating the influence of predoctoral publications on obtaining a postdoctoral position, as well as studies of the effect of postdoctoral publications on future faculty status, are warranted.

Another limitation of the current study is that it was conducted at only one institution, CU AMC. For context, as of 2018, CU AMC is ranked 23rd out of 431 U.S. Higher Education Institutions in terms of NIH funding received, as per NIH RePORTER (National Institutes of Health, 2018). In terms of R&D expenditures of institutions as ranked by the National Science Foundation, in 2016 (most recent data available) CU AMC was ranked 50th out of 900 (National Science Foundation & National Center for Science and Engineering Statistics, 2018b). The school is classified as an R2 Doctoral University (Higher Research Activity) in The Carnegie Classification of Institutions of Higher Education (2015). Further studies will be necessary to determine how generalizable the current findings are for graduates of institutions that differ from CU AMC in terms of research funding, focus, or types of doctorates awarded.

Interestingly, non-first-author publication record did not predict faculty status in any of the models. The importance of non-first-author publications on future academic success (e.g., job placement, promotions, etc.) is debatable, with one study even finding that middle author publications were potentially detrimental (Van Dijk, Manor & Carey, 2014). On the other hand, the presence of middle author publications is strong evidence of a desire to collaborate and may be indicative of technical knowledge. Nonetheless, the results of the current study suggest that middle author publication record during graduate school has little impact on future faculty status.

## Conclusions

In conclusion, the current study supports first-author publication record during graduate school as a potential predictor of future career outcome. Findings also suggest importance of publication impact factors, with higher impact factors also contributing to faculty status predictions. Given the myriad responsibilities graduate students must balance, including coursework, data collection, and often teaching, the current findings suggest that it is nonetheless important for mentors to strongly encourage and support predoctoral first-author publications, particularly in high-impact journals, if graduate students express a desire to obtain an eventual faculty position. That non-first-author publications were not predictive of career outcomes may suggest that focusing efforts toward first-author publications, and waiting until students are more established before concentrating on middle-author publications, may be a useful strategy to manage time constraints. At this point, however, these suggestions are only speculative, with further investigation needed to better understand how predoctoral publication record influences future career outcomes. Additional work is also necessary to understand how sex affects the impact of publication record on future faculty status.

It is useful to consider results from the present study in the context of the bigger picture of the academic job market. It is clear that the number of graduating PhD students far exceeds the capacity of the academic market. As such, many students remaining in academia find themselves in lengthy postdoctoral positions that may delay and/or hinder the development of their independent research careers (National Institutes of Health, 2012; Alberts et al., 2014). Given this, it is not only helpful for students and educators to better understand factors contributing to academic faculty placement, but also to address the larger issue that the number of available faculty positions is not sufficient to accommodate those who desire them, regardless of productivity or skill. As such, in addition to addressing pathways to academic faculty positions, it is important that students receive sufficient education regarding non-academic career paths before and during their PhD program. As suggested by Alberts et al. (2014), large-scale changes will ultimately be necessary to create a more sustainable system, such as limiting the number of graduate student and/or postdoctoral positions, increasing the ratio of staff scientists to trainees in research labs, and fundamental shifts in scientific funding paradigms. These types of changes also align with recommendations for supporting the next generation of biomedical and behavioral sciences researchers in the recent National Academies of Sciences, Engineering, and Medicine report (National Academies of Sciences, Engineering, and Medicine, 2018). The report recommends that research institutions collect and disseminate data on demographics and outcomes of pre- and post-doctoral researchers, enhance career guidance counseling for postdoctoral researchers, require formal training for postdoctoral mentors, and increase staff scientist positions. The report also calls for NIH to place a cap on the number of years of postdoctoral support allowed on NIH research grants, to increase support for early career researchers, and to work to promote diversity and inclusion at the junior faculty level. It is hoped that efforts such as these to improve transparency and increase the quality of information available will not only be helpful to individuals considering or beginning a career in science, but will also facilitate fundamental shifts in scientific funding and research institutions to encourage the success of the next generation of scientists.

## Supplemental Information

10.7717/peerj.5707/supp-1 Supplemental Information 1 Box-and-whisker plot of the probability of obtaining a faculty position as a function of weighted first-author publication count, divided by quartile.

## Additional Information and Declarations

### Data Availability

The following information was supplied regarding data availability: The raw data used in the analyses for this manuscript was provided by the University of Colorado Denver Office of the Registrar and supplied for peer review but because it is still possible to identify the participants even with de-identified data, the raw data cannot be published with the article.
