# Supplementary figures and images for: Predicting academic career outcomes by predoctoral publication record
Source: PeerJ. 2018 Oct 4;6:e5707. doi: 10.7717/peerj.5707 (PMC6174868; doi:10.7717/peerj.5707)

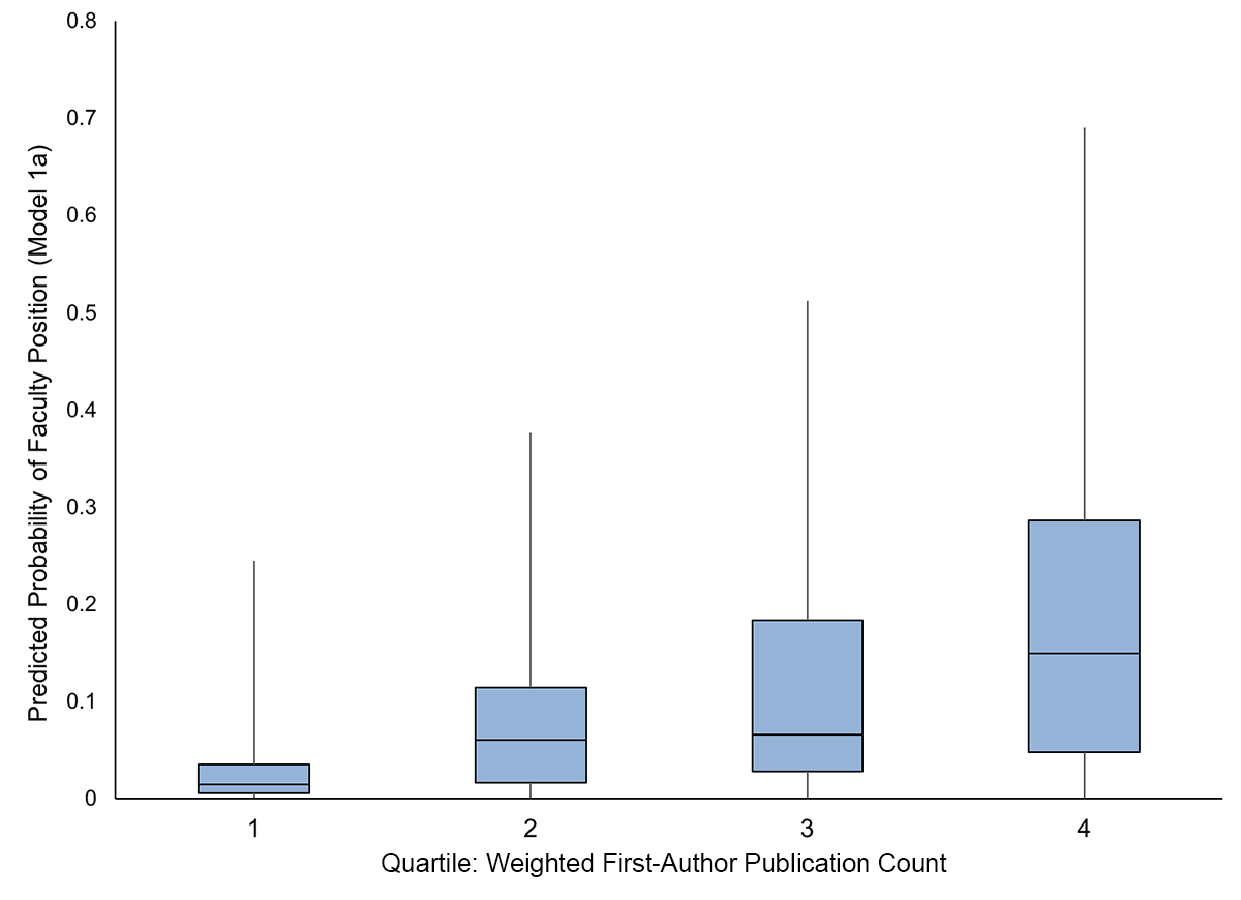

Supplement: Supplemental Information 1 [file peerj-06-5707-s001.png]
